# Essential Roles of Efferent Duct Multicilia in Male Fertility

**DOI:** 10.3390/cells11030341

**Published:** 2022-01-20

**Authors:** Mohammed Hoque, Eunice N. Kim, Danny Chen, Feng-Qian Li, Ken-Ichi Takemaru

**Affiliations:** 1 Molecular and Cellular Biology Graduate Program, Stony Brook University, Stony Brook, NY 11794, USA; hoque.mohammed@stonybrook.edu (M.H.); eunice.kim.2@stonybrook.edu (E.N.K.); 2 Department of Pharmacological Sciences, Stony Brook University, Stony Brook, NY 11794, USA; danny.chen@stonybrook.edu (D.C.); feng-qian.li@stonybrook.edu (F.-Q.L.)

**Keywords:** cilia, multiciliated cells, efferent ducts, spermatogenesis, fertility

## Abstract

Cilia are microtubule-based hair-like organelles on the cell surface. Cilia have been implicated in various biological processes ranging from mechanosensation to fluid movement. Ciliary dysfunction leads to a plethora of human diseases, known as ciliopathies. Although non-motile primary cilia are ubiquitous, motile multicilia are found in restricted locations of the body, such as the respiratory tract, the oviduct, the efferent duct, and the brain ventricles. Multicilia beat in a whip-like motion to generate fluid flow over the apical surface of an epithelium. The concerted ciliary motion provides the driving force critical for clearing airway mucus and debris, transporting ova from the ovary to the uterus, maintaining sperm in suspension, and circulating cerebrospinal fluid in the brain. In the male reproductive tract, multiciliated cells (MCCs) were first described in the mid-1800s, but their importance in male fertility remained elusive until recently. MCCs exist in the efferent ducts, which are small, highly convoluted tubules that connect the testis to the epididymis and play an essential role in male fertility. In this review, we will introduce multiciliogenesis, discuss mouse models of male infertility with defective multicilia, and summarize our current knowledge on the biological function of multicilia in the male reproductive tract.

## 1. Introduction

Cilia are microtubule-based, tiny hair-like organelles that extend from the apical surface of many different cell types [1,2,3]. Extensive work over the last few decades has revealed that cilia regulate a wide range of biological processes during development and adult homeostasis. Cilia are broadly classified into two types according to their microtubule composition: non-motile primary cilia with a 9 + 0 microtubule arrangement and motile multicilia with a 9 + 2 arrangement. The primary cilia in the embryonic node are a notable exception to this rule [4,5]. Nodal cilia, which are responsible for the establishment of the left–right body axis, have a 9 + 0 microtubule arrangement but show motility. The ciliary axoneme, which is composed of microtubule bundles with associated proteins, is surrounded by a specialized ciliary membrane that is continuous with the plasma membrane but has distinct properties with unique lipid and receptor compositions [6,7]. Primary cilia are present on many different cell types in the human body and play key roles in mechanosensation, photoreception, and various intracellular signaling pathways, including hedgehog (Hh), platelet-derived growth factor (PDGF), and G-protein-coupled receptor (GPCR) pathways [1,2]. In contrast, multicilia are found in limited locations in the human body, such as epithelial cells lining the respiratory tract, the female and male reproductive tracts, and the brain ventricles. Multiciliated cells (MCCs) contain dozens to hundreds of motile cilia that beat in a whip-like motion. The synchronous motility of multicilia is achieved by dynein motors in an ATP-dependent manner. The force generated by the ciliary beating typically drives directional fluid flow, for example, to clear airway mucus and debris, transport ova from the ovary to the uterus [8], and circulate cerebrospinal fluid in the brain [9]. Sperm flagella are more complex, singular motile cilia that are important for swimming and fertilization [10,11].

Cilia are assembled from mother centriole-derived basal bodies. In contrast to the daughter centriole, the mother centriole harbors accessory structures, including subdistal and distal appendages. The distal appendages (also called “transition fibers” at the ciliary base) are critical for the recruitment of small vesicles and subsequent docking of basal bodies to the plasma membrane [12,13,14,15]. Most ciliary proteins are imported from the cell body via polarized vesicle trafficking from the Golgi complex or endosomes [16,17]. The extension of a cilium and its maintenance require intraflagellar transport (IFT), a bidirectional transport system that tracks along the axonemal microtubules [18,19]. Although the mode of centriole generation differs, the formation of both primary cilia and multicilia follows a similar pathway as discussed in more detail below [20,21].

Given the ubiquitous presence of cilia and their important roles in embryonic development and adult homeostasis, it is not surprising that genetic defects in the structure and function of cilia are associated with a host of human disorders, collectively known as ciliopathies [2,3,22]. Dysfunctional primary cilia have been linked to various diseases and syndromes, such as polycystic kidney disease (PKD), Joubert syndrome (JBTS), and Bardet–Biedl syndrome (BBS) [3,23,24]. Their clinical features are variable but include situs inversus, polydactyly, retinal degeneration, intellectual disability, obesity, and cystic lesions of the kidney, liver, and pancreas. On the other hand, defects in multicilia are most prominently associated with primary ciliary dyskinesia (PCD) [25,26,27,28]. PCD is a rare genetic disorder, affecting approximately 1:10,000–40,000 individuals. To date, over 50 causative genes have been reported, including various axonemal components and regulatory factors of axonemal assembly and MCC differentiation [11,27,29]. PCD is characterized by recurrent respiratory infections, situs inversus, infertility, and more rarely hydrocephalus. Male infertility and sub-fertility are also common manifestations of PCD. Since sperm tail axonemes are structurally similar to motile cilia, malformations of sperm tails are commonly associated with PCD [11]. However, the exact effect of the gene mutations on the formation of sperm flagella remains poorly defined.

## 2. Differentiation of Multiciliated Cells (MCCs)

**Figure 1:**
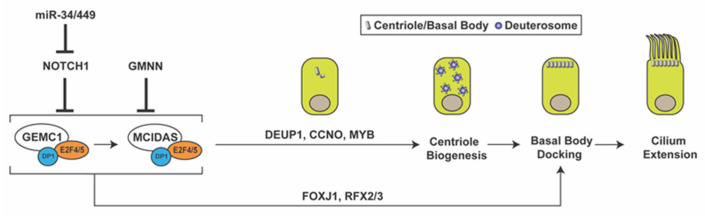
Multiciliated cell differentiation. Key factors and events for MCC differentiation are depicted. Repression of Notch signaling by miR-34/miR-449 triggers MCC differentiation. GEMC1 acts in a complex with E2F4/5 and DP1 to turn on MCIDAS and other ciliary genes necessary for centriole amplification, basal body docking, and cilium elongation. GMNN functions as an inhibitor of GEMC1 and MCIDAS. GMNN, geminin; GEMC1, geminin coiled-coil domain-containing protein 1; DEUP1, deuterosome assembly protein 1; CCNO, cyclin O; FOXJ1, forkhead box J1.

MCC differentiation is orchestrated by a coordinated cascade of regulators, including signaling pathways and transcription factors (Figure 1). Previous studies have demonstrated that the inhibition of the Notch and bone morphogenic protein (BMP) signaling pathways triggers a regulatory network of genes involved in the MCC fate determination of developing epithelia [30,31,32,33]. The upstream mechanisms that repress the Notch and BMP pathways are not fully understood. However, the microRNA clusters *miR-34/miR-449* have been shown to play essential roles at multiple levels in multiciliogenesis by destabilizing target mRNAs [34,35]. The miR-34/miR-449 family includes the functionally redundant miRNA clusters *miR-34b/c* and *miR-449a/b/c*, which are highly expressed in multiciliated tissues. In particular, miR-449 has been shown to promote MCC differentiation by directly repressing Notch1 and its ligand Delta-like 1 (DLL1) in progenitor cells [35,36,37].

Downstream of Notch inhibition, the MCC differentiation gene regulatory network is controlled by the coiled-coil Geminin family proteins Geminin (GMNN), Geminin coiled-coil domain-containing protein 1 (GEMC1), and MCIDAS (also known as multicilin or IDAS) [38,39,40,41,42,43,44,45,46]. Both GEMC1 and MCIDAS are master regulatory proteins that are necessary and sufficient for MCC differentiation [43,47]. Upon forming a ternary complex with the cofactor DP1 and transcription factors E2F4/5, GEMC1 is able to activate expression of *MCIDAS* and other downstream ciliary genes. MCIDAS also acts as a transcriptional activator by forming a complex with DP1 and E2F4/5 to stimulate gene expression essential for centriole biogenesis. Early multiciliogenesis factors that are downstream of GEMC1 and MCIDAS include deuterosome assembly protein 1 (DEUP1), MYB, cyclin O (CCNO), forkhead box J1 (FOXJ1), and RFX2/3 [41,43,44,45,48]. p73, another transcription factor downstream of MCIDAS, was found to activate expression of *FOXJ1*, *RFX2/3*, and *miR-34b/c* and nearly 50 other ciliary genes in mouse tracheal MCCs [49,50]. While the role of Geminin in MCC differentiation is less understood, it has been shown to act as an inhibitor of GEMC1 and MCIDAS function [40,44].

During MCC differentiation, the biogenesis of hundreds of centrioles occurs through two different pathways: the canonical centriolar pathway, which utilizes pre-existing centrioles for new centriole generation, and the de novo deuterosome-mediated pathway [51,52]. Although a small fraction of centrioles is produced via the canonical centriolar pathway, the majority are thought to be amplified from deuterosomes. Deuterosomes are electron-dense, fibrogranular structures that contain several proteins required for centriole amplification, such as DEUP1 and coiled-coil domain-containing 78 (CCDC78) [48,53,54]. Although research on the origins and molecular components of deuterosomes is still ongoing, it has been reported that deuterosomes are nucleated from existing centrioles or spontaneously synthesized in the cytoplasm [55,56,57]. A recent study questioned the need for the deuterosome in MCC centriole amplification, since MCCs lacking deuterosomes are able to amplify the correct number of centrioles with normal kinetics [58].

**Figure 2:**
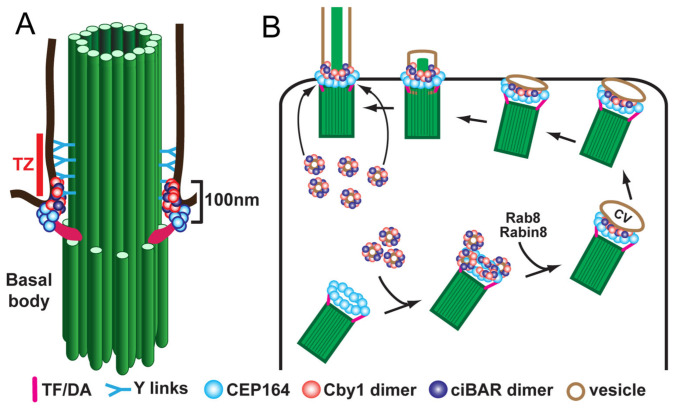
Model for localization (**A**) and function (**B**) of Cby1 and its associated proteins in airway MCCs. Cby1 clusters at the ciliary base as a ring with a diameter of 300 nm and a height of 100 nm [13]. Cby1 and its interactors are essential for the efficient docking of basal bodies to the apical membrane. TZ, transition zone; TF, transition fiber; DA, distal appendage; CV, ciliary vesicle.

Following release from the deuterosome or centrosomal centriole, the nascent centrioles migrate to the apical cell surface, dock at the plasma membrane, and mature into basal bodies to initiate cilium assembly. For efficient basal body docking to occur, small vesicles are recruited to the distal appendages of centrioles and then fuse to form a larger membranous cap called the ciliary vesicle [13,53,59]. Subsequently, the ciliary vesicle flattens and forms a bilayer membranous sheath around the developing ciliary axoneme that merges with the cell membrane, allowing the elongating cilium to protrude from the apical cell surface. Vesicular trafficking during ciliogenesis has been studied mainly using mammalian cultured cells with primary cilia. In early stages of ciliogenesis, the fusion of small vesicles into the ciliary vesicle is mediated by the EPS-15 homology domain-containing (EHD) family of membrane-shaping proteins, EHD1 and EHD3 [52,60]. The small GTPase Rab11 then recruits and activates Rabin8, a guanine nucleotide exchange factor (GEF) for Rab8, in the vicinity of the centrosome. Rabin8, in turn, promotes the recruitment and local activation of Rab8 to facilitate ciliary membrane growth and vesicular transport of ciliary proteins into the cilium [61,62]. Rab8 and Rabin8 also interact with the distal appendage protein CEP164 (also known as NPHP15), which acts as a molecular bridge between the mother centriole and components of the ciliary membrane assembly machinery [12]. Our group demonstrated that, in airway MCCs, the 15-kDa coiled-coil protein Chibby1 (Cby1) plays an important role in ciliary vesicle formation and basal body docking (Figure 2) [13,63]. *Cby1^−/−^* mice exhibit chronic upper airway infection, due to a markedly decreased number of multicilia and a complete absence of mucociliary clearance activity [63]. Cby1 and its interacting proteins localize to the base of cilia (Figure 2A). Cby1 is recruited to the distal appendages of basal bodies through its physical interaction with CEP164 and interacts with Rabin8 to facilitate the efficient assembly of ciliary vesicles (Figure 2B) [13,64]. Cby1 exists in a complex with the membrane remodeling proteins, Chibby1-interacting Bin/Amphiphysin/Rvs (BAR) domain-containing 1 and 2 (ciBAR1 and ciBAR2; previously known as FAM92A and FAM92B, respectively) [64,65]. Coexpression of ciBAR1 or ciBAR2 with Cby1 in mammalian cultured cells induces the formation of globular and tubular membrane structures, suggesting that the Cby1/ciBAR complex facilitates ciliogenesis through regulation of membrane-remodeling processes.

The docking and planar polarization of basal bodies are controlled by the interplay between the planar cell polarity (PCP) pathway and the underlying actin and microtubule cytoskeleton [66,67,68,69,70,71]. The PCP pathway is a noncanonical Wnt pathway and has emerged as a critical regulator of MCC differentiation. In MCCs, the orientation of basal bodies is established via rotational polarity at the apical cell surface to enable synchronous beating of motile multicilia on each cell. PCP components localize to opposing membrane domains at the proximal and distal sides of cells [68,69]. The core PCP components include the evolutionarily conserved transmembrane proteins Frizzled, Van Gogh-like (Vangl, Van Gogh in *Drosophila*), Celsr (Flamingo in *Drosophila*) and cytoplasmic proteins Dishevelled, Prickle, and Inversin/Diversin (Diego in *Drosophila*). Mutations in the core PCP components lead to basal body docking defects and random orientation of basal bodies and ciliary beating in MCCs.

The extension and maintenance of cilia depend on the IFT machinery [18,19]. Along the cilium, anterograde IFT movement is mediated by kinesin-2 motors (from base to tip), whereas retrograde transport is powered by cytoplasmic dynein-2 (from tip to base). Fully developed cilia are highly dynamic and constantly undergo turnover with continuous transport of proteins and lipids in and out of cilia [72,73].

## 3. Male Reproductive Tract and Passage of Spermatozoa through Efferent Ducts

**Figure 3:**
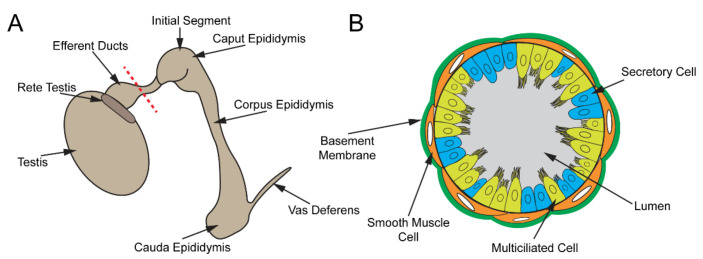
Male reproductive tract. (**A**) Cartoon schematic of the male reproductive tract. Sperm are produced in the seminiferous tubules of the testis, collected in the rete testis, and transported to the epididymis via EDs, where they mature. Mature sperm are stored in the cauda epididymis and released through the vas deferens. (**B**) Depiction of a cross-section of the ED along the red dashed line in (**A**). The ED epithelium consists of MCCs and secretory cells.

The male reproductive tract is composed of several highly convoluted tubular segments, each of which plays a crucial role in the development of fully functional spermatozoa (sperm) (Figure 3A). The testis, consisting of long convoluted seminiferous tubules, is the site of spermatogenesis [74,75,76]. From the testis, the spermatozoa are collected in the rete testis and are transported into the epididymis via the efferent ducts (EDs) [77,78]. The EDs contain specialized MCCs that prevent sperm from aggregating [79]. In the epididymis, sperm continue to mature and are finally stored in the cauda epididymis for eventual release via the vas deferens [80,81].

The development of spermatozoa takes place in the testis. The seminiferous tubules consist of multiple cell types, including spermatogenic cells, Sertoli cells, and peritubular myoid cells. The spermatogenic cycle occurs in waves, and different regions of the seminiferous tubules contain spermatogenic cells at varying steps of differentiation. Sertoli cells are nurse cells that aid spermatogenesis by anchoring developing spermatids to the seminiferous tubule epithelium and establishing the blood–testis barrier [82]. Peritubular myoid cells are smooth muscle-like cells that surround the seminiferous tubules and provide structural rigidity to the seminiferous tubules and contract to push spermatozoa and fluid into the rete testis.

Spermatogenesis is initiated by the asymmetric cell division of spermatogonial stem cells. Following several rounds of mitotic cell divisions, primary spermatocytes go through two rounds of meiosis to give rise to spermatids [75]. Spermatids then undergo spermiogenesis, in which round spermatids transform into elongated spermatozoa with a flagellum and a species-specific head shape [74]. Upon completion of spermiogenesis, the spermatozoa appear morphologically mature but lack motility and the capacity to fertilize an egg [83]. Sertoli cell secretions release spermatozoa into the lumen of the seminiferous tubules and coordinated contractions of peritubular myoid cells propel sperm into the rete testis [79].

Once collected in the rete testis, sperm transit into the EDs. The EDs originate from mesonephric tubules and are critical for concentrating the seminiferous fluid by reabsorbing water and ions and transporting sperm into the epididymis [78,84,85,86]. Three-dimensional reconstruction of mouse EDs revealed that EDs are 22.5 µm in mean radius and 81.9 mm in length [87]. Sperm transport through the EDs occurs within 45 min in rats [88]. The number of EDs in the mice varies from 2 to 5 [87,89,90,91]. In golden Syrian hamsters, there are six ducts [92]. In humans and other large mammals, EDs are more numerous and feed into the epididymis at various sites, whereas EDs in rodents collect into a singular duct (known as the common duct) prior to entering the epididymis [93].

The ED epithelium contains MCCs and nonciliated cells that are surrounded by a single layer of smooth muscle cells (Figure 3B). Several studies have shown that both MCC and nonciliated cell populations in the EDs are required for proper sperm movement into the epididymis [77,94]. The abundance of different cell populations varies along the length of the EDs. The proximal end of EDs contains more nonciliated cells than MCCs, whereas the ducts close to the epididymis have substantially more MCCs [95]. Approximately 90% of the seminiferous fluid is thought to be reabsorbed by the nonciliated cells in the EDs. Absorption of water is driven by aquaporin (AQP) water channels [96,97]. Aside from water, nonciliated cells are also responsible for the reabsorption of low molecular weight solutes and proteins [84,85,98,99,100]. These processes are primarily governed by active transport. Passive water transport, on the other hand, is partly regulated by estrogen [94,101]. While estrogen is primarily known for its role in the development and maintenance of the female reproductive system, the estrogen receptors α (*ESR1*) and β (*ESR2*) are expressed abundantly in the EDs [101,102,103]. ESR1 regulates expression of sodium/hydrogen exchanger-3 (*NHE3* or *SLC9A3*) and the aquaporins *AQP1* and *AQP9*, which modulate sodium and fluid homeostasis within the ED lumen, respectively [94,104,105]. Accordingly, loss of *ESR1* in mice leads to defects in fluid reabsorption and ED development [104,106,107]. The EDs of *ESR1^−/−^* mice were found to be substantially enlarged with low counts of cauda epididymal sperm, due to fluid accumulation [106]. To date, the biological role of ESR2 in the male reproductive tract remains unknown. The smooth muscle cells contract to push sperm toward the epididymis. 

The epididymis, which is divided into four regions (initial segment, caput, corpus, and cauda epididymis), serves as the site of sperm concentration, maturation, and storage (Figure 3A) [91]. In the initial segment, fluid absorption continues to occur, albeit to a much lesser degree than in the EDs [100,107]. Throughout the rest of the epididymis, various secretory and absorptive processes set up a unique microenvironment that increases the fertilization competency of the sperm. As such, early studies have reported that mouse sperm isolated from the caput yielded a fertilization rate of only 11% *in vitro*, compared to 33% when cauda epididymal sperm were used [108]. Furthermore, normal development to the blastocyst stage was observed in 48% of those successfully fertilized with cauda sperm, while only 8% of caput sperm embryos developed to a blastocyst. After passage through the corpus epididymis, sperm finally reach the cauda epididymis, where they are stored for release via the vas deferens.

## 4. Roles of Multiciliated Cells in Efferent Ducts

As the conduit between the testis and the epididymis, the EDs are essential for male fertility. As spermatozoa leave the testis, they are concentrated 20-fold by passing through the EDs [98,106]. The ED is the only portion of the entire male reproductive tract that harbors MCCs. Like typical motile multicilia, ED multicilia have a 9 + 2 microtubule organization [77]. However, the tips of ED multicilia show twisting of the axonemal microtubules and are capped with a structure known as the ciliary crown that consists of small claw-like extensions [77]. Ciliary claws are also found in other multiciliated tissues [109] and are approximately 5 nm in width, although their functional significance is currently unknown. As observed in the airway, oviduct, spinal cord, and brain ventricles, it has been proposed that the sweeping motion of multicilia in the EDs propel sperm from the rete testis to the epididymis. However, recent findings suggest that multicilia in the ED beat in a whip-like rotary motion that stirs the contents of the lumen to prevent sperm aggregation [77,79]. Without functional MCCs in EDs, sperm aggregate, ultimately leading to obstruction of the EDs. This may cause fluid retention and back pressure in seminiferous tubules, resulting in degeneration of the seminiferous epithelium and infertility. Ligation of the EDs, which simulates ED obstruction, recapitulated these findings, supporting the idea that loss of multicilia leads to a blockage in the EDs [110,111]. Several mouse models with defective multicilia have been reported to show extensive sperm occlusion in the ED, which prevents sperm transit from the testis to the epididymis, resulting in male infertility (Table 1) [112,113,114,115].

## 5. Mouse Models with Defective Multicilia in Efferent Ducts 

The E2F family of transcription factors consist of 8 members in mammals (E2F1-8) and regulate the G1/S transition of the cell cycle [116,117,118]. Among them, E2F4 and E2F5 play key roles in MCC differentiation [38]. During MCC differentiation, GEMC1 and MCIDAS form a complex with E2F4/5 and DP1 transcription factors to induce expression of critical MCC genes, such as *CCNO* and *FOXJ1* [38,40,119]. *E2F4^−/−^* mice suffer from chronic rhinitis and associated opportunistic bacterial infections, and more than 85% of them die by 3 weeks of age [120]. Consistent with these phenotypes, *E2F4^−/−^* mice show an absence of MCCs from the entire airway epithelium and the epithelium of submucosal glands in the paranasal sinuses. Importantly, expression of *FOXJ1* is abolished in the *E2F4^−/−^* respiratory epithelium, indicating that E2F4 acts upstream of FOXJ1. *E2F5^−/−^* mice develop hydrocephalus around 3–4 weeks after birth, and most die within 6 weeks of age [121]. This suggests that E2F5 is necessary for ependymal MCC differentiation in brain ventricles, although the status of multicilia in *E2F5^−/−^* mice have not been investigated. E2F4 and E2F5 play overlapping and redundant roles, since simultaneous deletion of *E2F4* and *E2F5* leads to neonatal lethality [116]. Interestingly, conditional deletion of *E2F4* in the EDs in combination with heterozygous mutation of *E2F5* (*E2F4^fl/fl^/E2F5^+/−^;Villin-Cre*) results in defective MCCs and male infertility [113]. Analysis of the male reproductive tract revealed an accumulation of sperm in the EDs, expansion of the seminiferous tubule lumen, dilation of the rete testis, and little to no sperm in the epididymis. Furthermore, MCCs in the EDs failed to differentiate and form multicilia, and nonciliated cell development was also altered with reduced expression of estrogen receptor-α (*ESR1*) and aquaporin 1 (*AQP1*). Collectively, these data clearly demonstrate that E2F4 and E2F5 are important for differentiation of MCCs in the EDs and, hence, male fertility.

GEMC1 and MCIDAS belong to the Geminin family of cell cycle and transcriptional regulators and are essential for MCC differentiation. *GEMC1^−/−^* mice are significantly smaller than their wild-type counterparts, lack multicilia in the brain ventricle, respiratory system, and reproductive tract, and display common ciliopathy features, such as hydrocephalus and infertility in both sexes [40,44]. Transmission electron microscopy or immunofluorescence staining of the adult tracheal epithelium revealed no identifiable MCCs in *GEMC1^−/−^* mice. Moreover, transcriptional profiling of the trachea and oviduct of *GEMC1^−/−^* mice using microarrays showed reduced expression levels of many ciliary genes, including *MCIDAS*, *FOXJ1*, and *CCNO* [40]. In agreement with this, GEMC1 directly activates the upstream regulatory sequence of *MCIDAS* and *FOXJ1* [40]. Similarly, *MCIDAS^−/−^* mice are severely runted and show hydrocephalus and infertility without any detectable multicilia [45]. In contrast to *GEMC1^−/−^* mice, MCCs in *MCIDAS^−/−^* mice express *FOXJ1* and other MCC transcription factors. This suggests that MCIDAS is not required for the specification of the MCC lineage but necessary for subsequent differentiation processes, such as centriole replication. GEMC1 and MCIDAS, as well as their downstream target *CCNO*, are essential for male fertility in mice [115]. CCNO (cyclin O) plays a key role in centriole amplification during MCC differentiation [122,123]. *CCNO* expression is restricted to MCCs, and *CCNO^−/−^* mice exhibit characteristic features of MCC dysfunction, including hydrocephalus, mucociliary clearance deficit, and infertility [123,124]. *GEMC1^−/−^*, *MCIDAS^−/−^*, and *CCNO^−/−^* male mice are infertile, due to defective multicilia in the EDs [115]. These mice show similar phenotypes, such as thinning of seminiferous tubule epithelia, dilation of the rete testis, sperm accumulation in the EDs, and lack of sperm in the epididymis. Notably, homozygous frameshift mutations in *MCIDAS* have been recently reported in an infertile male patient showing no sperm in the ejaculate (azoospermia) with almost complete loss of multicilia in the EDs [125]. 

Similarly, deletion of the functionally related microRNA clusters *miR-34/miR-449* in mice yields the common phenotypes associated with defective multicilia, including male infertility. However, single knockout mice are viable and fertile with no apparent phenotypes [35,79,126,127,128,129,130]. A spermatogenic cell-specific *miR-34/miR-449*-double conditional mouse line, generated by mating with a *Stra8-Cre* driver mouse, shows normal male fertility [79], indicating that spermatogenesis proceeds normally in the absence of miR-34/miR-449. Closer analyses of *miR-34b/c^−/−^/miR-449^−/−^* male mice revealed loss of multicilia in the EDs, leading to sperm clumping, rete testis dilation, and ED occlusion [79]. MCC-specific inactivation of *miR-34/miR-449* using a *FOXJ1-Cre* driver phenocopies the ED deficits of the germline *miR-34b/c^−/−^/miR-449^−/−^* male mice, reinforcing the view that loss of multicilia in the EDs is the primary cause of their male infertility. Yuan *et al.* also reported that multicilia in the EDs uniquely show whip-like beating with constant changes in direction and generate turbulence to maintain immotile sperm in suspension, which are further pushed into the epididymis by the peristaltic contractions of the ED smooth muscle [79].

Dynein axonemal heavy chain 5 (DNAH5) is a component of the outer dynein arm of multicilia and essential for ciliary beating. Mutations in *DNAH5* represent the most frequent cause of PCD and account for 15–2% of PCD cases [131]. A recent study reported that mice deficient for *DNAH5* (*Mdnah5^mut/mut^*) exhibit accumulation of sperm in the EDs, rete testis dilation, and infertility, due to dysmotility of ED multicilia that lack the outer dynein arms [112]. In contrast, sperm isolated from *Mdnah5^mut/mut^* mice show normal flagellar ultrastructures and motility. Consistent with these findings, DNAH5 is only detectable along multicilia but not in sperm flagella. Notably, similar to the phenotypes of *Mdnah5^mut/mut^* mice, human PCD patients with loss-of-function mutations in *DNAH5* manifest decreased sperm counts (oligozoospermia) and dilation of the epididymal head but have normal sperm motility. Thus, this study clearly establishes that loss of motility of multicilia in the EDs is sufficient to cause ED obstruction and male infertility.

**Figure 4:**
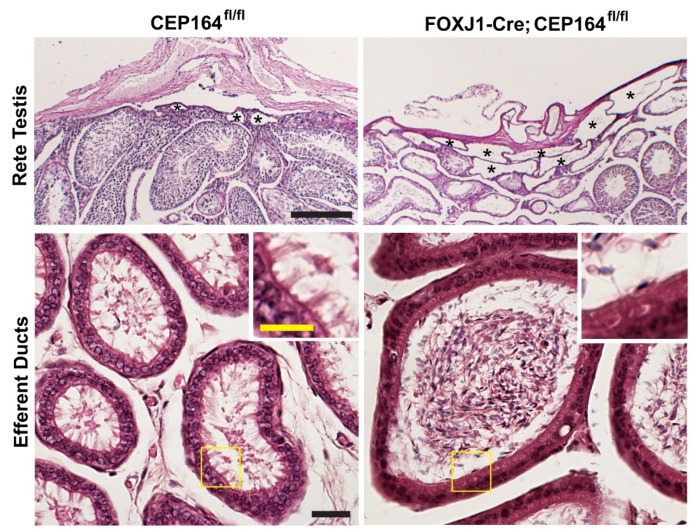
Rete testis dilation and sperm aggregation in the EDs of the *FOXJ1-Cre;Cep164^fl/f^^l^* mouse model. PAS staining of the rete testis (top) and H&E staining of EDs (bottom) from adult mice. Asterisks indicate the tubules of the rete testis. Scale bar, 200 μm. The boxed areas in yellow are magnified in the insets to show multicilia. Note the lack of multicilia in the EDs of *FOXJ1-Cre;Cep164^fl/f^^l^* mice. Scale bars, 20 μm and 10 μm (magnified images).

CEP164 is a distal appendage protein that is essential for ciliogenesis (Figure 2) [64,132,133]. CEP164 recruits small vesicles to basal bodies to facilitate anchoring of basal bodies to the apical cell surface, thereby promoting cilium assembly [12,64]. Our group conditionally eliminated *CEP164* in MCCs using a *FOXJ1-Cre* mouse line (*FOXJ1-Cre;CEP164^fl/fl^*). *FOXJ1-Cre;Cep164^fl/f^^l^* mice show a profound loss of airway, ependymal, and oviduct multicilia and develop hydrocephalus and male infertility. About 20% die around weaning age with severe hydrocephalus. Basal body recruitment of CEP164 interactors Cby1 and ciBAR1, and ciBAR2 was severely reduced in primary cultures of tracheal MCCs in the absence of CEP164. More recently, we have demonstrated that their male infertility is attributable to an almost complete loss of multicilia in the EDs (Figure 4) [114]. *FOXJ1-Cre;Cep164^fl/f^^l^* mice displayed extensive dilation of the rete testis and aggregation of sperm in EDs (Figure 4), along with aggregation of sperm in the seminiferous tubules. The sperm aggregation in the seminiferous tubules has not been reported in other mouse models with impaired MCCs, suggesting that the ED defects in *FOXJ1-Cre;CEP164^fl/fl^* mice are more severe or that CEP164 plays a role in biological processes distinct from MCC differentiation. Loss of *CEP164* also resulted in basal body docking defects, as evidenced by accumulation of basal bodies in the cytoplasm of the ED epithelium. These studies further support the notion that dysfunctional multicilia in the EDs can be a cause of male infertility, independent of sperm flagellar formation. The role of CEP164 in spermatogenesis remains to be explored. Mutations in *CEP164* have been identified in a host of ciliopathy patients including nephronophthisis, BBS, PCD, and oral-facial-digital syndrome [134,135,136,137,138], but male reproductive phenotypes have not been reported.

## 6. Conclusions

MCCs in the EDs play an essential role in male fertility by mediating the swift transport of sperm from the testis into the epididymis. While the differentiation mechanisms and key roles of MCCs in the respiratory, ependymal, and oviduct epithelia have been extensively studied, the important function of MCCs in the EDs has only recently been recognized. Data from mouse models and human patients as described above provide compelling evidence that multicilia in the EDs play essential roles in male fertility. Impaired MCC functions in EDs present with sperm aggregation, obstruction of sperm transport, and degeneration of germ cells in the testis, ultimately leading to infertility. Although more genetic studies of infertile men are required, multicilia in the EDs may be considered as a critical factor when diagnosing male infertility.

## Figures and Tables

**Table 1:** Mouse models and human patients with defective ED multicilia.

Mouse	Ciliary Function	Male Reproductive Phenotypes	Human	Ref
*E2F4^fl/fl^/E2F5^+/−^;Villin-Cre*	TFs; early MCC differentiation	Infertility; Dilation of STs and RT; Sperm accumulation in EDs; Little to no sperm in CE; Reduced AQP1 expression in EDs		[113]
*GEMC1^−/−^*	TF; early MCC differentiation	Infertility; Dilation of STs and RT; SC degeneration; Sperm accumulation in EDs; No sperm in CE		[115]
*MCIDAS^−/−^*	TF; early MCC differentiation	Infertility; Dilation of STs and RT; SC degeneration; Sperm accumulation in EDs; No sperm in CE	Azoospermia; Loss of ED multicilia	[115]
*CCNO^−/−^*	Cyclin O; deuterosome formation and centriole amplification	Infertility; Dilation of STs and RT; Sperm accumulation in EDs; No sperm in CE		[115]
*miR-34b/c^−/−^/miR-449^−/−^*	microRNAs; early MCC differentiation	Infertility; Dilation of STs and RT; Sperm granuloma in RT; Sperm accumulation in EDs		[79]
*Mdnah5^mut/mut^*	ODA component; ciliary beating	Reduced sperm counts; Dilation of RT; Sperm accumulation in EDs; Little to no sperm in CE	Oligozoospermia; Normal sperm motility	[112]
*FOXJ1-Cre;Cep164^fl/fl^*	Distal appendage component; recruitment of Cby1/ciBAR complexes	Infertility; Reduced sperm counts; Dilation of STs and RT; Sperm accumulation in EDs; Sperm aggregation in STs and CE		[114]

TF, transcription factor; MCC, multiciliated cell; ODA, outer dynein arm; STs, seminiferous tubules; RT, rete testis; ED, efferent duct; CE, cauda epididymis; SC, Sertoli cell.

## Data Availability

Not applicable.
